# Comparing transthoracic echocardiography, 2D and 4D flow cardiovascular magnetic resonance for quantitative valve assessment after percutaneous pulmonary valve implantation

**DOI:** 10.1093/ehjimp/qyag054

**Published:** 2026-03-29

**Authors:** Marguerite E Faure, Joao G Carvalho, Annemien E van den Bosch, Jeroen M Wilschut, Tim ten Cate, Anthonie L Duijnhouwer, Jolien W Roos-Hesselink, Ricardo P J Budde, Alexander Hirsch

**Affiliations:** Department of Radiology and Nuclear Medicine, Erasmus MC, Room Rg-419, PO Box 2040, 3000 CA, Rotterdam, The Netherlands; Department of Radiology, AZ Monica, Antwerp, Belgium; Department of Radiology and Nuclear Medicine, Erasmus MC, Room Rg-419, PO Box 2040, 3000 CA, Rotterdam, The Netherlands; Department of Radiology, Centro Hospitalar Universitário de Santo António, Porto, Portugal; Department of Cardiology, Cardiovascular Institute, Thorax Center, Erasmus MC, Room Rg-419, PO Box 2040, 3000 CA, Rotterdam, The Netherlands; Department of Cardiology, Cardiovascular Institute, Thorax Center, Erasmus MC, Room Rg-419, PO Box 2040, 3000 CA, Rotterdam, The Netherlands; Department of Cardiology, Radboud University Medical Center, Nijmegen, The Netherlands; Department of Cardiology, Radboud University Medical Center, Nijmegen, The Netherlands; Department of Cardiology, Cardiovascular Institute, Thorax Center, Erasmus MC, Room Rg-419, PO Box 2040, 3000 CA, Rotterdam, The Netherlands; Department of Radiology and Nuclear Medicine, Erasmus MC, Room Rg-419, PO Box 2040, 3000 CA, Rotterdam, The Netherlands; Department of Cardiology, Cardiovascular Institute, Thorax Center, Erasmus MC, Room Rg-419, PO Box 2040, 3000 CA, Rotterdam, The Netherlands; Department of Radiology and Nuclear Medicine, Erasmus MC, Room Rg-419, PO Box 2040, 3000 CA, Rotterdam, The Netherlands; Department of Cardiology, Cardiovascular Institute, Thorax Center, Erasmus MC, Room Rg-419, PO Box 2040, 3000 CA, Rotterdam, The Netherlands

**Keywords:** percutaneous pulmonary valve implantation, transthoracic echocardiography, cardiovascular magnetic resonance, phase contrast, 4D flow

## Abstract

**Aims:**

To compare transthoracic echocardiography (TTE), 2D and 4D flow cardiovascular magnetic resonance (CMR) for the evaluation of valve function and flow measurements after percutaneous pulmonary valve implantation (PPVI).

**Methods and results:**

In this multicentre exploratory observational study, 29 patients who underwent PPVI were planned for TTE and CMR with 2D and 4D flow measurements after PPVI. Peak velocity was measured with all techniques. Net and regurgitating flow measurements were measured with CMR distal to the transcatheter pulmonary valve (TPV). Median peak velocity of the TPV with TTE was 2.5 (2.0–3.1) m/s, with 2D flow CMR 2.3 (2.0–3.2) m/s, and with 4D flow 2.3 (2.0–3.0) m/s. There was a good correlation for the peak velocity of the TPV between TTE and 2D flow CMR (*r* = 0.68, *P* < 0.001) and a moderate correlation for peak velocity between TTE and 4D flow CMR (*r* = 0.56, *P* = 0.003), and between 2D flow and 4D flow CMR (*r* = 0.56, *P* = 0.006). For peak velocity measurements, no proportional bias was observed between the three imaging techniques. For the CMR flow measurements, a strong correlation was observed between 2D and 4D flow CMR, for both net flow (*r* = 0.87, *P* < 0.001) and regurgitation fraction (r = 0.96, *P* < 0.001) of the TPV. Net flow through the left pulmonary artery correlated strongly between 2D and 4D flow CMR (*r* = 0.85, *P* < 0.001), and there was a good correlation for the right pulmonary artery (*r* = 0.65, *P* < 0.01). For net flow measurements of the TPV, we found an underestimation of flow measurements at higher flow rates with 4D flow CMR, compared to 2D flow CMR.

**Conclusion:**

TTE, 2D flow CMR, and 4D flow CMR are all feasible and reliable imaging techniques after PPVI, concordant in predominantly mild-to-moderate stenosis. These findings strengthen the use of 2D and 4D flow CMR as a tool for post-PPVI assessment, despite stent-induced artefacts, although reliability in more advanced valve dysfunction remains uncertain.

## Introduction

Patients with congenital heart diseases involving the right ventricular outflow tract (RVOT) often undergo multiple surgical interventions early in life. Tetralogy of Fallot (TOF) is the most common congenital heart disease to affect the RVOT.^1^ Also, patients with a diseased aortic valve are sometimes transplanted with the patient’s own pulmonary valve, followed by replacing the pulmonary valve with a donor valve (most often a homograft is used; i.e. the Ross procedure). Unfortunately, RVOT dysfunction due to pulmonary regurgitation and/or stenosis often occurs in these patients after the initial operation, and multiple re-interventions are frequently required. Surgical replacement of the pulmonary valve or conduit is often needed for managing RVOT or conduit dysfunction to prevent the development of irreversible right ventricular injury.^2^ However, surgical repair becomes technically more challenging with each subsequent procedure. Percutaneous pulmonary valve implantation (PPVI) offers a minimally invasive alternative to patients with RVOT dysfunction and is an effective procedure associated with reduced hospital length of stay and quick recovery.^3–5^ In PPVI, a stented bioprosthetic valve is placed in the pulmonary annulus or right ventricular (RV) conduit. The native valve or conduit remains in place, and the leaflets are left in the open position adjacent to the transcatheter pulmonary valve (TPV). In patients with an existing valve prosthesis, TPV can be placed into the existing frame of the bioprosthetic valve (called valve-in-valve). Imaging plays a crucial role in both preprocedural planning and postprocedural follow-up.^6^ Nevertheless, a common challenge in clinical practice remains the timing of imaging and which modality is best suited for a given patient scenario. Moreover, artefacts of the stent frame are a limiting factor in post-PPVI imaging. Post-PPVI imaging studies remain scarce, and data on 4D flow cardiovascular magnetic resonance (CMR) measurements in TPV are even more scarce. Therefore, the aim of this study was to compare three different imaging techniques to evaluate post-PPVI measurements of valve function and flow measurements: transthoracic echocardiography (TTE), 2D flow CMR, and 4D flow CMR.

## Methods

### Study design and population

In this multicentre exploratory observational study, patients who had a history of PPVI and were ≥18 years were prospectively invited to undergo TTE and CMR at the same day. This was done by reviewing the list of patients who underwent a PPVI in the Erasmus Medical Center or Radboud University Medical Center hospital between 2007 and 2019. Exclusion criteria were estimated glomerular filtration rate <30 mL/min, claustrophobia, cardiac pacemaker/internal cardiac defibrillator, unwillingness to be informed about unrequested imaging findings, and mentally incapacitated adults. The medical ethical committee approved the study and all patients provided informed consent (COVER, MEC-2020–0257).

### Imaging protocols and analysis

TTE was performed on an iE33 or Epiq (Philips Medical Systems) ultrasound system equipped with an X5-1 matrix transthoracic probe. In all patients, a comprehensive 2-dimensional (2D) TTE in harmonic and Color Doppler imaging was performed. Valvular regurgitation and stenosis were evaluated according to the European Association of Echocardiography recommendations.^7^ Peak transpulmonary velocity was measured, both subvalvular and valvular. The regurgitation severity of the TPV was scored using a semi-quantitative algorithm described previously by our group using diastolic flow reversal in the pulmonary artery and pressure half time.^8^

CMR was performed on a clinical 1.5T scanner (SIGNA Artist, GE Healthcare, Milwaukee, WI, USA). For functional imaging, electrocardiogram-gated cine balanced steady-state free-precession images were obtained during repeated breath-holds in standard long-axis views. Contiguous short-axis slices were acquired, covering the entire left ventricle (LV) and RV from base to apex. Typical scan parameters were one slice per breath-hold, slice thickness 6 mm, inter-slice gap 4 mm, flip angle 65°, ASSET 2, field of view (FOV) 32–38 cm, phase FOV 75–100%, acquired matrix 200 × 280 with 30 reconstructed phases per cardiac cycle. Standard 2D phase contrast sequences were performed at four levels: (i) at the sinotubular junction perpendicular to the aorta, (ii) between the distal edge of the PPVI and the pulmonary bifurcation perpendicular to the pulmonary artery, (iii) left pulmonary artery, and (iv) right pulmonary artery. Scan parameters were FOV 31–38 cm, phase FOV 75–100%, slice thickness 7 mm, matrix size 192 × 160, flip angle 20°, ASSET 2, views per segment 4 to 6 based on patients’ heart rate, and 30 reconstructed cardiac phases. The standard velocity encoding (VENC) value was set at 180 cm/s and gradually increased up to 500 cm/s if necessary. No background phase correction was applied for 2D flow measurements. Directly after contrast administration (Gadovist, 0.2 mmol/kg, administered as a bolus), 4D flow data were acquired in the axial plane including the heart and major thoracic vessels during free-breathing using retrospective ECG gating with respiratory motion compensation. Typical scan parameters were FOV 32–38 cm, phase FOV 70–100%, matrix size 160 × 160, flip angle 15°, Kat-ARC (or HyperKat) factor 8, number of excitations 4, and 30 reconstructed phases per cardiac cycle. The flow-encoding scheme was symmetric four-point, VENC was set at 180 cm/and increased if necessary up to 500 cm/s. This was optimized depending on available echocardiography data and 2D flow settings. The median VENC of the 2D flow for TPV measurements was 250 (25th to 75th percentile, 208–280) cm/s and of the 4D flow 190 (180–263) cm/s. All CMR images were analysed semi-automatically using dedicated post-processing software (Qmass software version 8.1, Qflow 8.1, and Qflow4D 1.1, Medis Medical Imaging, Leiden, the Netherlands). Ventricular volumes, mass, and ejection fraction were measured on the short-axis cine images using standard methods, including the papillary muscles and trabeculations in the blood volume. All volumes and masses were indexed by body surface area. Antegrade, retrograde, and net flow were measured on the 2D phase contrast images in the four described planes. Automatic border detection was used. These contours were reviewed and adapted manually when necessary for each cardiac phase using the magnitude and phase contrast images. Concerning the 4D flow analysis, automated velocity offset correction was applied (3rd order, 25% static tissue correction). The four described planes for 2D flow measurements were also reconstructed on the 4D flow images using multiplanar reconstructions and the same parameters were measured. Moderate or greater valve regurgitation was defined as a regurgitation fraction ≥20% for 2D and 4D flow CMR. Severe stenosis was defined as a peak velocity of ≥4.0 m/s.

### Statistical analysis

IBM SPSS Statistics 28.0 software was used for analysis. QQ-plots and Shapiro–Wilk test were performed to evaluate data distribution. Categorical data are presented as number with percentage, continuous data as mean ± standard deviation (SD), or median and 25th to 75th percentile as appropriate. TTE and CMR measurements were compared by paired-samples T-test when data were normally distributed, or Wilcoxon signed-rank test in case of non-normally distributed data. The Pearson correlation coefficient, with 95% confidence interval, was used to measure the correlation between valve haemodynamics with TTE and 2D and 4D flow CMR. We defined a strong Pearson correlation coefficient as *r* ≥ 0.8, a good correlation as *r* ≥ 0.6, and a moderate correlation was defined as r ≥ 0.5. *R* < 0.5 was considered a weak correlation.^9,10^ Bland–Altman plots were constructed to assess agreement between methods.^11^ Mean difference was calculated and the limits of agreement between two measurements were determined as the mean of the difference ± 1.96 SD. Additionally, the coefficient of variation was provided to compare the dispersion of two variables. *P*-values <0.05 were considered significant.

## Results

In total, 89 patients underwent PPVI between 2007 and 2019. During follow-up, six patients died, in four patients, PPVI was not successful, and two patients underwent valve explantation afterward. Four patients did not meet the inclusion criteria. Therefore, 73 patients were invited to participate in the study. Due to the COVID-19 pandemic at the start of inclusion and the precarious health status of these patients, a relatively large number of patients were unwilling to come to the hospital. Finally, 29 patients (40%) gave informed consent and underwent TTE and CMR.

Baseline characteristics of the cohort are presented in *Table 1*. The mean age of the patients at the time of imaging was 39.8 ± 11.7 years and 79% (23/29) were male. The median time between PPVI and post-procedural imaging was 6.4 (4.0–9.0) years. In 26 patients, a Melody valve (Medtronic, Minneapolis, MN, USA) was implanted, and in three patients a SAPIEN transcatheter heart valve (Edwards Lifesciences, Irvine, CA, USA).

**Table 1:** Baseline characteristics and cardiovascular magnetic resonance data of the patients who underwent percutaneous pulmonary valve implantation

	*n* = 29
Patient age (years)	39.8 ± 11.7
Male	23 (79%)
Body mass index (kg/m^2^)	23 ± 3.9
New York Heart Association class I/II	23 (79%)
Underlying pathology	
Tetralogy of Fallot	10 (35%)
Ross procedure for aortic valve disease	12 (41%)
Transposition of the great arteries	3(10%)
Pulmonary atresia with VSD	2 (7%)
Congenital pulmonary stenosis	2 (7%)
Medications pre-PPVI	
βeta-blocker	5 (17%)
ACE inhibitor/angiotensin receptor blocker	2 (7%)
Diuretics/mineralocorticoid receptor antagonists	0 (0%)
Direct oral anticoagulants or coumarin derivates	3 (10%)
Aspirin	2 (7%)
RVOT landing zone	
Pulmonary homograft conduit	22 (76%)
Native	1 (3%)
Bioprosthetic (Melody-in-Melody)	4 (14%)
Other	2 (7%)
Indication PPVI	
Valve stenosis	18 (62%)
Valve insufficiency	5 (17%)
Combination	4 (14%)
Unknown	2 (7%)
Implanted valve type	
Melody valve	26 (90%)
SAPIEN valve	3 (10%)
Median time between PPVI—Imaging (years)	6.4 (4.0–9.0)
Cardiovascular Magnetic Resonance	
Left ventricle	
End-diastolic volume (mL/m^2^)	98 ± 20
End-systolic volume (mL/m^2^)	44 ± 13
Stroke volume (mL/m^2^)	53 ± 10
Ejection fraction (%)	55 ± 7
Mass (g/m^2^)	64 ± 13
Right ventricle	
End-diastolic volume (mL/m^2^)	111 ± 44
End-systolic volume (mL/m^2^)	58 ± 38
Stroke volume (mL/m^2^)	53 ± 12
Ejection fraction (%)	51 ± 12

Data are presented as number (percentage), mean ± standard deviation or median (25th to 75th percentile). ACE = angiotensin-converting enzyme; PPVI = percutaneous pulmonary valve implantation; RVOT = right ventricular outflow tract; VSD = ventricular septum defect.

The underlying pathology was, in most cases, a Ross procedure for aortic valve disease (41%) or TOF (35%). In 76% (22/29) of patients, RVOT landing zone consisted of a pulmonary homograft conduit. The main reason for PPVI was progressive pulmonary stenosis (62%), 17% had only pulmonary insufficiency, 14% had both relevant stenosis and insufficiency, and in 7% the indication of PPVI was unknown.

**Figure 1:**
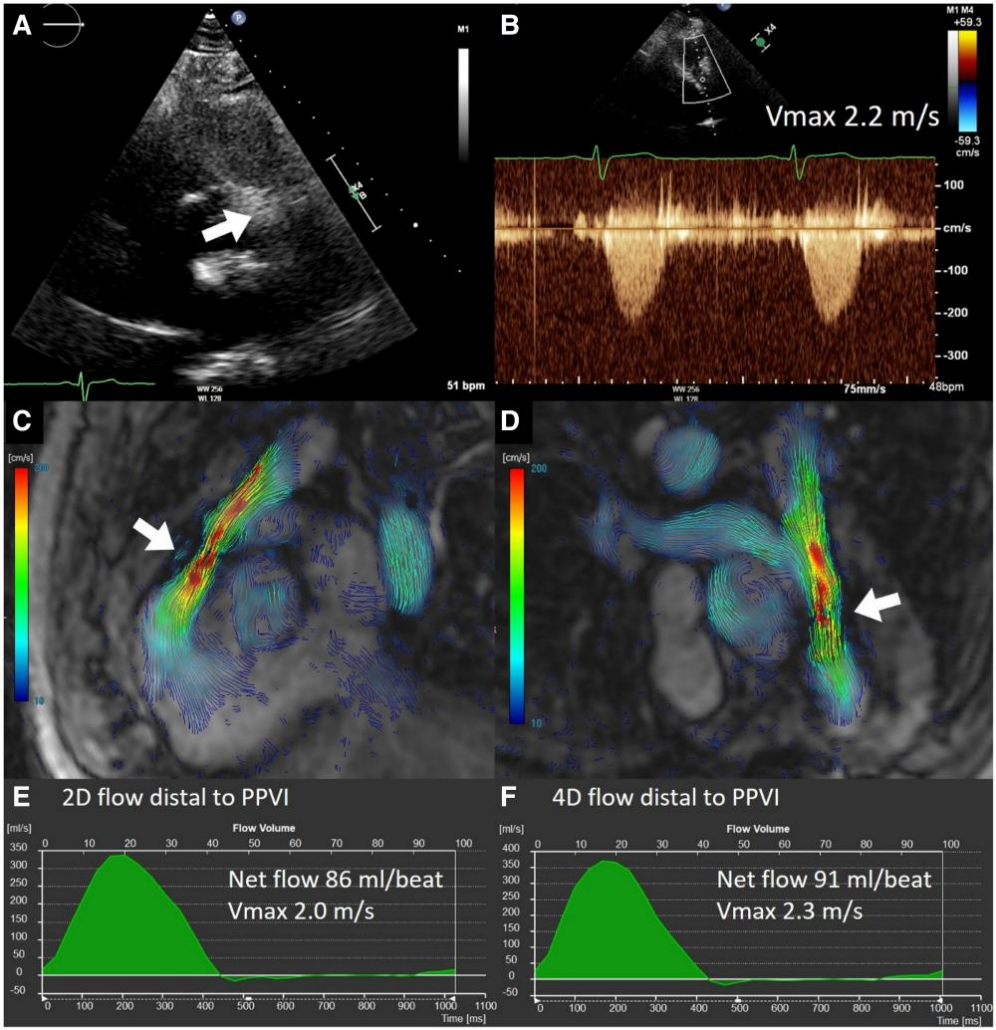
Example of a transthoracic echocardiogram and cardiovascular magnetic resonance in a patient after implantation of a percutaneous pulmonary valve implantation. (*A*) parasternal view of the right ventricular outflow tract with the transcatheter pulmonary valve in situ (Melody valve, white arrow), (*B*) continuous wave Doppler signal with maximal velocity of 2.2 m/s. (*C* and *D*) 4D flow cardiovascular magnetic resonance images of the right ventricular outflow tract and pulmonary artery and branches. (*E* and *F*) corresponding 2D and 4D flow measurements distal to the valve. The maximum velocity was 2.0 and 2.3 m/s, respectively, with a net flow of 86 and 91 mL/beat without any regurgitation.

**Figure 2:**
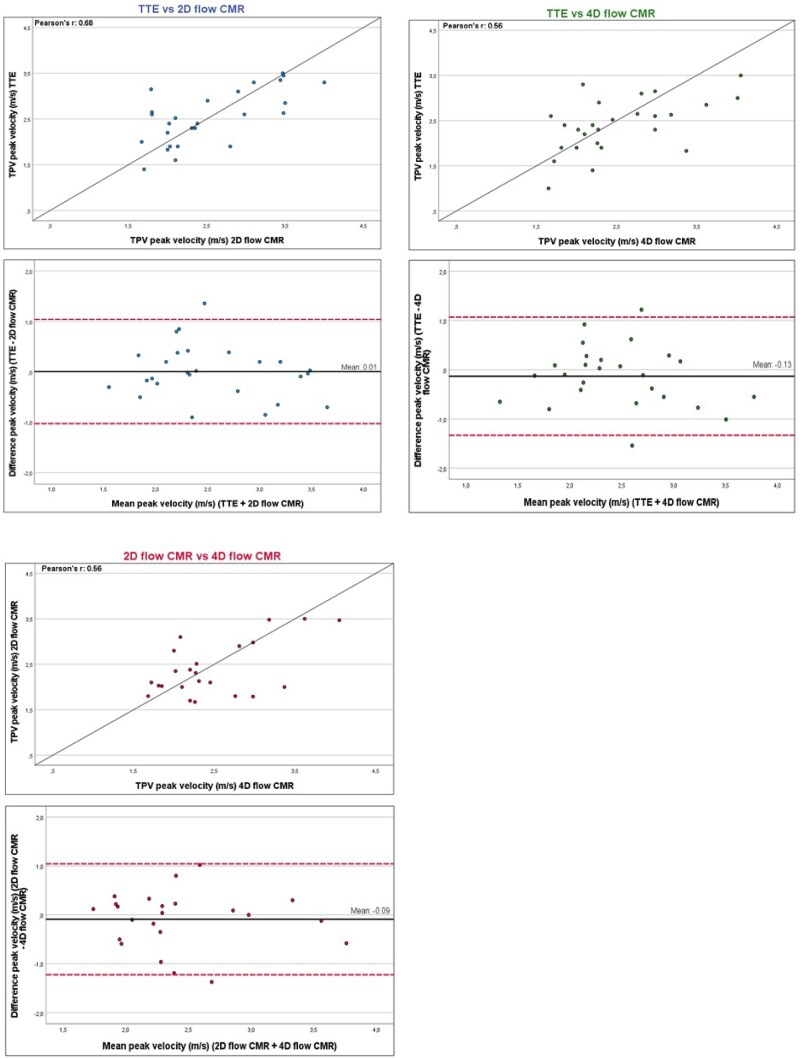
Inter-modality agreement for peak transcatheter pulmonary valve (TPV) velocity. Agreement between transthoracic echocardiography (TTE), 2D flow cardiovascular magnetic resonance (CMR), and 4D flow CMR. Blan–Altman plots and identity line (solid black line) for TTE vs. 2D flow CMR (blue), TTE vs. 4D flow CMR (green), and 2D flow vs. 4D flow CMR (red). Dashed red lines in Blant–Altman plots indicate ±1.96 standard deviation.

An example of a TTE and CMR in patient after PPVI is presented in *Figure 1*. The results of TTE, compared to 2D flow and 4D flow CMR, are presented in *Table 2*. Inter-modality agreement of pulmonary valve measurements for the different imaging modalities are shown in *Table 3*. In all 29 patients, peak velocity through the TPV could be measured with TTE. In both 2D and 4D CMR, there were three patients in whom peak velocity measurements were unreliable or technically insufficient. Median peak velocity across TPV by TTE was 2.5 (2.0–3.1) m/s, using 2D flow CMR 2.3 (2.0–3.2) m/s, and by 4D flow 2.3 (2.0–3.0) m/s. There was a good correlation for the peak velocity across TPV between TTE and 2D flow CMR (*r* = 0.68, *P* < 0.001) and a moderate correlation for peak velocity between TTE and 4D flow CMR (*r* = 0.56, *P* = 0.003), and between 2D flow and 4D flow CMR (*r* = 0.56, *P* = 0.006) (*Table 3*). In *Figure 2*, the correlations and Bland–Altman plots are shown for the comparisons. No clear over- or underestimation was observed between the three imaging techniques. The number of patients with a severe stenosis (≥4.0 m/s) over the TPV was very limited (no severe stenosis was measured with TTE, only one when measured with 2D flow CMR and two patients with 4D flow CMR). This means no conclusions can be made about the reliability of 2D and 4D flow to accurately measure high grade stenosis of the TPV. Correlations for peak velocity in left and right pulmonary artery were both weak (*r* = 0.26 and *r* = 0.48, respectively).

**Table 2:** Valve parameters post percutaneous pulmonary valve implantation stratified by imaging modality

	TTE	2D flow CMR	4D flow CMR
**Transcatheter pulmonary valve**	*n* = 29	*n* = 26	*n* = 26
Peak velocity (m/s)	2.5 (2.0–3.1)	2.3 (2.0–3.2)	2.3 (2.0–3.0)
Net flow (mL/beat)	—	86 (76–99)	85 (72–95)
Regurgitation fraction (%)	—	2 (1–5), (range 0–25)	2 (0–4), (range 0–25)
Moderate or greater regurgitation	5 (17%)	3 (12%)^a^	2 (8%)^a^
**Left pulmonary artery**		*n* = 19	*n* = 25
Net flow (mL/beat)	—	40 (30–48)	44 (35–52)
**Right pulmonary artery**		*n* = 22	*n* = 26
Net flow (mL/beat)	—	45 (36–52)	45 (38–59)

Data presented as number (percentage), median (25th–75th percentile) unless otherwise stated. CMR = cardiovascular magnetic resonance; TTE = transthoracic echocardiography.

^a^Defined as a regurgitation fraction ≥20% on CMR.

**Table 3:** Inter-modality agreement of pulmonary valve measurements for the different imaging modalities

	Pearson’s r (95% CI)	*P*-value	Mean difference	Lower LOA	Upper LOA	COV (%)
**TTE vs. 2D flow CMR**						
Peak TPV velocity (m/s)	0.68 (0.42–0.86)	<0.001	0.01	−1.03	1.04	26
**TTE vs. 4D flow CMR**						
Peak TPV velocity (m/s)	0.56 (0.20–0.80)	0.003	−0.13	−1.33	1.07	24
**2D flow vs. 4D flow CMR**						
Peak TPV velocity (m/s)	0.56 (0.11–0.82)	0.006	−0.09	−1.22	1.04	27
Net flow TPV (mL/beat)	0.87 (0.71–0.95)	<0.001	5.24	−20.11	30.59	22
Regurgitation fraction TPV (%)	0.96 (0.86–0.99)	<0.001	0.18	−3.67	4.04	44
Net flow LPA (mL/beat)	0.85 (0.53–0.96)	<0.001	−2.49	−14.85	9.87	32
Peak LPA velocity (m/s)	0.26 (0.00–0.66)	0.312	−0.13	−0.98	0.72	27
Net flow RPA (mL/beat)	0.65 (0.23–0.84)	<0.001	−5.34	−25.97	15.30	28
Peak RPA velocity (m/s)	0.48 (0.05–0.76)	0.032	−0.68	−1.81	0.44	34

CI = confidence interval; CMR = cardiovascular magnetic resonance; COV = coefficient of variation; LOA = limit of agreement; LPA = left pulmonary artery; RPA = right pulmonary artery; TTE = transthoracic echocardiography; TPV = transcatheter pulmonary valve.

*P*-value <0.05 was considered significant.

**Figure 3:**
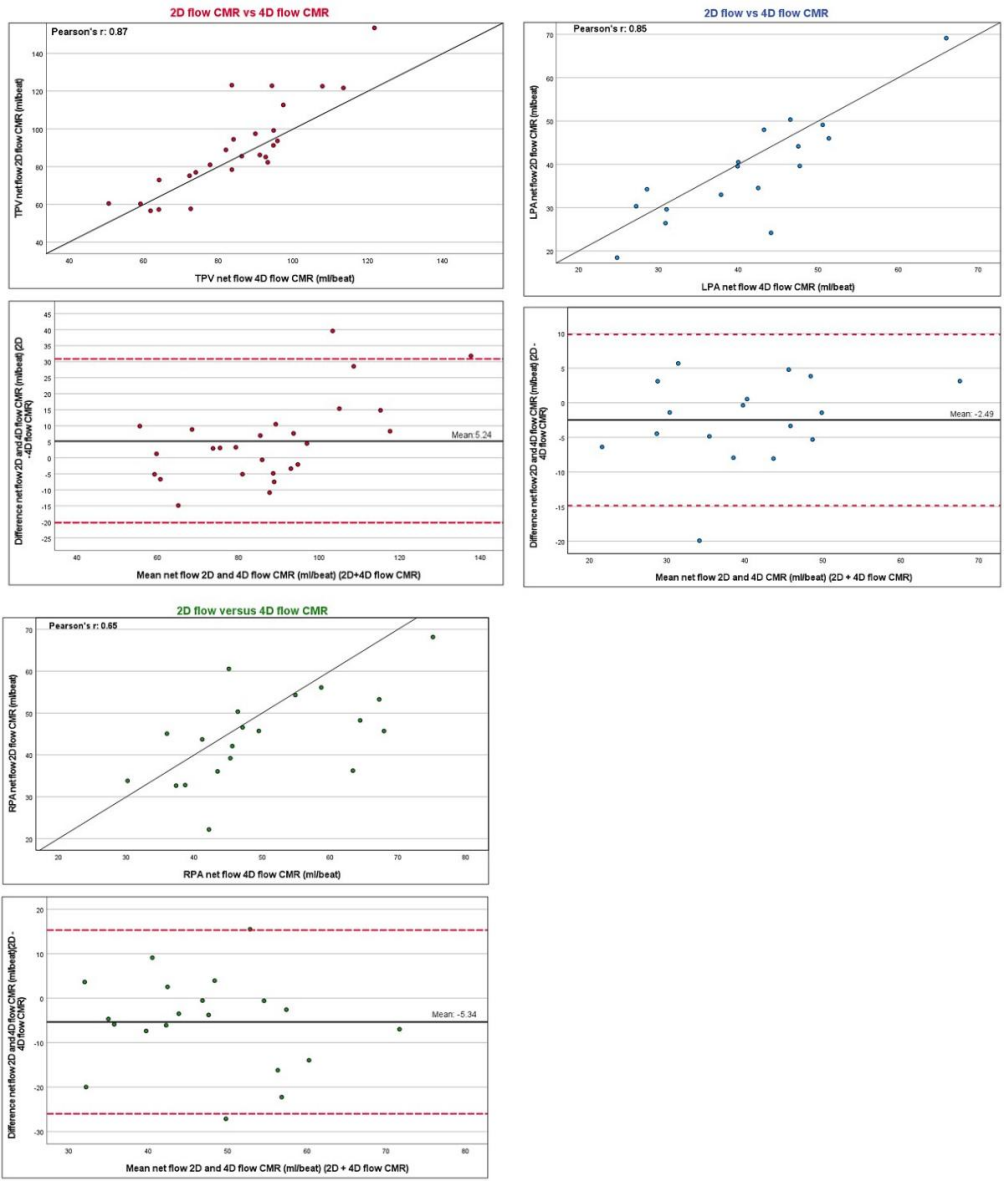
Inter-modality agreement for transcatheter pulmonary valve (TPV) and left and right pulmonary artery (LPA and RPA) net flow. Agreement between 2D flow cardiovascular magnetic resonance (CMR) and 4D flow CMR. Blant–Altman plots and identity line (solid black line) for 2D flow vs. 4D flow CMR. Dashed red lines in Blant–Altman plots indicate ±1.96 standard deviation.

Median net flow in the pulmonary artery just distal from the TPV was 86 (76–99) mL/beat for 2D flow CMR and 85 (72–95) mL/beat for 4D flow CMR (*Table 2*) with a strong correlation when comparing these two techniques (r = 0.87, *P* < 0.001) (*Figure 3*). The mean difference was 5 mL/beat. At higher flow rates, there was an underestimation of flow measurements with 4D flow CMR. Also, a strong correlation was found when comparing the regurgitation fraction of the TPV with 2D and 4D flow CMR (*P* = 0.96, *P* < 0.001). The mean difference was 0%. Moderate or greater TPV regurgitation was found in five (17%) patients with TTE, in three (12%) with 2D flow CMR, and three (8%) with 4D flow CMR. Due to these small numbers, little can be said regarding the reliability of 2D and 4D flow CMR to accurately measure moderate- to high-grade regurgitation in TPV. Of the 26 patients with all three modalities, there were no patients with moderate-to-severe regurgitation on 2D or 4D flow CMR who were graded as none/mild regurgitation on TTE. One patient with moderate-to-severe regurgitation on TTE was graded as none/mild regurgitation on 2D flow CMR, and two patients with moderate or greater regurgitation on TTE were graded as none/mild regurgitation on 4D flow CMR (see supplementary data online, *Table S1*).

Due to aliasing, in only 19 patients net flow could be measured in the left pulmonary artery with 2D flow CMR and in 22 patients the right pulmonary artery. For 4D flow CMR, these numbers were 25 and 26 patients, respectively. Median net flow volume in left pulmonary artery was 40 (30–48) mL/beat with 2D flow CMR and 44 (35–52) mL/beat with 4D flow CMR. For the right pulmonary artery, median net flow volume was 45 (36–52) mL/beat and 45 (38–59) mL/beat for 2D and 4D flow CMR, respectively. There was a strong correlation when comparing 2D and 4D flow CMR for net flow in the left pulmonary artery (*r* = 0.85, *P* < 0.001) and a good correlation for the net flow in the right pulmonary artery (*r* = 0.65, *P* < 0.01).

## Discussion

This study demonstrates that velocity and flow measurements are feasible with both 2D and 4D flow CMR after PPVI, with measurements performed distal to the TPV. For peak velocity measurements, we found a good correlation between 2D flow CMR and TTE and a moderate correlation between 4D flow CMR and TTE and between 2D and 4D flow CMR. For flow measurements after PPVI, we found a strong correlation between 2D and 4D flow CMR, for both net volume and regurgitation fraction of the TPV.

CMR is the current reference standard to evaluate RV volumes and function and pulmonary valve regurgitation, due to its ability to quantify volumes and flow. It is therefore widely implemented in the follow-up of patients with RVOT pathology. After PPVI however, CMR can be challenging due to stent artefacts.

Literature regarding the comparison of TTE and 2D and 4D flow CMR is rather scarce. 2D flow measurements by CMR are well established, robust and validated for flow quantification. Studies have demonstrated that 4D flow CMR also is a reliable tool for flow and velocity measurements with good reproducibility and low inter-observer variability and no significant difference in stroke volumes in the main and branch pulmonary arteries when comparing 2D and 4D flow CMR.^12–16^ In 2D flow CMR underestimation of the velocity can occur if the measurement is not performed perpendicular to the vessel or not at the position of maximum flow. The scan plane is planned during the scanning itself and cannot be changed during post-processing. In 4D flow CMR, the plane for analysis can be positioned retrospectively and with use of 3D anatomical data and visualization of the flow, the plane can be positioned exactly perpendicular to the vessel and the blood flow and therefore less prone to underestimation.^15,17^ In our study, however, we observed an underestimation of net flow by 4D flow compared to 2D flow CMR at higher flow rates, although we acknowledge this is based on only a few patients. Possible explanations include lower spatiotemporal resolution, intravoxel dephasing, signal loss near metallic stents, and acquisition acceleration. The lower temporal resolution of the 4D flow might have underestimated the flow. At higher flow rates, the temporal resolution of imaging may become insufficient, and the imaging sequence may miss rapid flow changes, particularly when blood velocity is high. Although we did not observe this phenomenon in our validation study, which used invasive flow measurements as a reference.^18^ Furthermore, although during post-processing of the 4D flow measurements, we carefully planned the measurements similarly to the 2D flow, the position might have influenced the flow measurements.^19,20^ Therefore, caution is warranted when interpreting 4D flow–derived net flow measurements in situations where high peak velocities or turbulent flow are expected, such as in the presence of residual pulmonary stenosis, significant regurgitation, or elevated cardiac output. 4D flow CMR results should be interpreted carefully when the measured net flow in the main pulmonary artery is inconsistent with other measurements, like systemic flow measurements.

When comparing 4D flow CMR with TTE, we must rely on studies regarding the aortic valve. Literature shows that there is a significant correlation for the peak velocity measurement through the aortic valve measured by 4D flow CMR and Doppler TTE.^21^ Importantly, it is noteworthy that although TTE is the first-line test for assessing aortic valve function, TTE has also limitations. The approximation of blood flow as a single streamline by continuous-wave Doppler TTE overestimates valvular pressure gradients compared to invasive measurements. In addition, the effective orifice area, used for regurgitation measurement, is calculated using the continuity equation, which includes many geometric and physiological assumptions.^22–24^ The assessment of pulmonary regurgitation by TTE has been largely qualitative and challenging. There is a tendency to underappreciate pulmonary regurgitation severity with TTE, and significant pulmonary regurgitation can be missed.^8,25^

After valve surgery, things become even more challenging, especially when metallic structures are used, which cause artefacts. After PPVI, however, data are scarce. In the few studies in which CMR was performed before and after PPVI, CMR has been demonstrated to outperform TTE in the evaluation of cardiac chambers, especially the RV, and is also, in this setting, considered a first-choice technique.^26^ More data are available for transcatheter aortic valve implantation. A meta-analysis regarding the use of TTE and CMR for the assessment of aortic regurgitation after transcatheter aortic valve implantation found that TTE generally correlates poorly with CMR for (paravalvular) aortic regurgitation assessment post-transcatheter aortic valve implantation, with a wide variation in the level of agreement. However, despite this significant discordance between the two imaging modalities over all grades of aortic regurgitation, TTE has a good ability to discriminate moderate or severe from mild or no aortic regurgitation.^27^

In post-PPVI CMR, due to the metallic structure of the TPV, there is a signal void in the valve area. Especially the Melody valve, which was the main implanted valve in this study, has a large signal void from the large strut and the use of pre-stenting. This mean pulmonary flow was not accurately measurable in the valve and was measured just distal to the stent. Our study shows a good correlation between TTE and 2D flow CMR findings and a moderate correlation between 4D flow CMR and TTE and between 2D and 4D flow CMR when looking at peak velocity of the TPV with a good inter-modality agreement. This suggests CMR can be used for pulmonary valve stenosis measurement after PPVI without any relevant underestimation of peak velocity of the TPV. However, in our study, only a limited number of patients had a severe stenosis or regurgitation of the TPV. Therefore, our outcomes are concordant in predominantly mild-to-moderate stenosis or regurgitation. It is still hard to conclude if CMR can measure high-grade stenosis accurately. Furthermore, Bland-Altman limits of agreement for peak velocity are relatively wide (±1.07–1.33 m/s for TTE vs. 4D flow CMR), which may be clinically relevant in borderline cases. Although correlations are generally acceptable, methods should be used with care near guideline-defined intervention thresholds. Also, correlations for peak velocity in LPA and RPA were both weak, which could suggest that peak velocity measurements are more sensitive to methodological limitations than flow quantification, particularly in smaller vessels. Flow measurements distal to the TPV show a strong correlation between 2D and 4D flow CMR with good inter-modality agreement. 2D flow CMR is widely used and is currently considered the reference standard for flow measurements. However, this technique is more complex and requires anatomical expertise. Furthermore, 2D flow CMR is time consuming both for acquisition and interpretation and thus generates a certain discomfort for the patients. In patients with repaired congenital heart defects, whose cardiac anatomy is modified by the surgical procedures in a way that varies from patient to patient, identifying the acquisition planes is even more challenging. Moreover, 2D flow CMR doesn’t allow the tracking of the valve plane, while 4D flow CMR does. Several authors demonstrated that the valve tracking method improves reliability and accuracy of flow measurement.^28,29^ These studies, however, are done in native valves. Because of the specific challenges of prosthetic valves in the pulmonary position, we did not use valve tracking and performed our measurements in a static plane. The strong correlation of flow measurements between 2D and 4D flow CMR in this study strengthens the use of 4D flow CMR for post-PPVI assessment. However, the inability to use dynamic valve tracking, and relatively moderate correlation between peak velocities compared to TTE, both temper the advantage of 4D flow over 2D flow CMR and TTE in this specific cohort. We didn’t show any clear superiority of 4D flow CMR over 2D flow CMR in our study.

The most important limitation of this study is the limited number of patients who were included, especially those with moderate-to-severe TPV stenosis or regurgitation. Probably, patients in relatively good health were willing to participate in the study, while those with complications may have been less likely to enrol, potentially introducing a selection bias. Also, due to the COVID-19 pandemic at the start of inclusion a relatively large number of patients were unwilling to come to the hospital, probably patients with worse health status. Due to the cross-sectional design of the study, patients with severe deterioration of the TPV were limited. However, only 6 of 89 patients died, and only 2 patients had valve explantation between implantation and the study date. Another limitation of the study is that we didn’t test for test-retest and intra- or interobserver variability. However, in previous studies, test-retest and interobserver agreement were good for calculating total flow and peak systolic velocity with 4D flow CMR.^16,30^ It is important to notice the differences between the two CMR techniques. In 2D flow CMR, the plane placement during acquisition can influence net flow and peak velocity measurements, whereas in 4D flow CMR, this analysis plane can be adjusted during post-processing within the 3D velocity dataset, introducing observer variability in the analysis phase instead of the acquisition phase.

## Conclusion

TTE, 2D and 4D flow CMR of the TPV are feasible after PPVI, despite difficulties concerning stent artefacts. Good correlation of peak velocity between TTE and 2D CMR demonstrates the possibility of using CMR in post-PPVI evaluation, in TPV with mild-to-moderate stenosis. 4D flow CMR has a strong correlation for both net and regurgitating volume measurements compared to 2D flow CMR and has the qualities to become a useful tool for post-PPVI assessment, allowing an overall assessment of cardiac anatomy and function coupled to complete flow analysis with unlimited post processing. Because our study group is relatively small and has only a limited number of patients with clinically significant valve dysfunction, further studies are needed.

## Supplementary Material

qyag054_Supplementary_Data

## Data Availability

The data underlying this article will be shared on reasonable request to the corresponding author.

## Lead author biography

Marguerite Elisabeth Faure completed her residency in Radiology in 2015 at the University hospital of Antwerp. In this year she followed a clinical fellowship at the department of cardiovascular radiology in the Erasmus Medical Center in Rotterdam to learn more in depth about cardiac MR imaging. These months in Rotterdam stimulated her to start research in the field of cardiac imaging and more specific in the field of prosthetic heart valve imaging. Since 2018 she works as a clinical radiologist with special focus on cardiovascular and breast imaging in AZ Monica Ziekenhuis in Deurne, Antwerp.
