# Supplementary material for: Comparing transthoracic echocardiography, 2D and 4D flow cardiovascular magnetic resonance for quantitative valve assessment after percutaneous pulmonary valve implantation
Source: Eur Heart J Imaging Methods Pract. 2026 Mar 29;4(1):qyag054. doi: 10.1093/ehjimp/qyag054 (PMC13075955; doi:10.1093/ehjimp/qyag054)
Supplement: qyag054_Supplementary_Data [file qyag054_supplementary_data.docx]

*Table S1: transcatheter pulmonary valve regurgitation assessed with TTE, 2D and 4D flow CMR*

|  | **2D flow CMR (n=26)** | | **4D flow CMR (n=26)** | |
| --- | --- | --- | --- | --- |
| **TTE** | None/mild regurgitation | Moderate/severe regurgitation | None/mild regurgitation | Moderate/severe regurgitation |
| None/mild regurgitation on TTE | 22 | 0 | 22 | 0 |
| Moderate/severe regurgitation on TTE | 1 | 3 | 2 | 2 |

*Numbers indicate the number of patients.*

*CMR = cardiovascular magnetic resonance; TTE = transthoracic echocardiography*
